# Informal sexual relationship-associated factors among young adult smartphone users in South of Iran: a cross-sectional study

**DOI:** 10.1186/s40359-023-01179-2

**Published:** 2023-04-25

**Authors:** Hassan Joulaei, Elahe Khaksar, Mohammad Ghorbani, Elham Rahmanipour, Nooshin Zarei, Zohre Foroozanfar

**Affiliations:** 1 Health Policy Research Center, Institute of Health, Shiraz University of Medical Sciences, Shiraz, Iran; 2 Shiraz University of Medical Sciences, Shiraz, Iran; 3 Faculty of Medicine, Mashhad University of Medical Sciences, Mashhad, Iran; 4 HIV/AIDS Research Center, Institute of Health, Shiraz University of Medical Sciences, Shiraz, Iran

**Keywords:** Smartphone, Sexual behavior, Young adult, Religious beliefs, Iran

## Abstract

**Background:**

With the unprecedented pace of modernization, risky sexual behaviors have become more frequent in developing countries, such as Iran. We aimed to assess the prevalence of informal sexual relationship (ISR) and factors associated with having ISR in young adult in Iran.

**Methods:**

This cross-sectional study was conducted on 414 young adult smartphone users in Iran, in 2019. Data was collected through an online questionnaire (including: ISR, socio-demographic variables, their use of social network, religious beliefs, personality, and loneliness). Logistic regression model was used to determine factors related to ISR.

**Results:**

A total of 152 (36.7%; 95% CI 32.1–45.6) participants reported having ISR. Finding an opposite-sex friend through a mobile app (OR = 2.59, 95% CI 1.34, 5.01), being currently sexually active (OR = 2.39, 95% CI 1.26, 4.56), higher scores of extroverted personality (OR = 1.13, 95% CI 1.01, 1.27), and closer relationship with parents (OR = 3.17, 95% CI 2.25, 8.02) were found to be associated with having ISR. Additionally, living in small cities rather than the provincial capital (OR = 0.23, 95% CI 0.10, 0.49) had a reverse association with having ISR.

**Conclusions:**

This study illustrated the high prevalence of ISR and its association with increased duration of internet and mobile app use. Innovative and multidisciplinary approaches could be recommended in this regard.

## Introduction

Sexual behaviors are described and compared in terms of social norms, and societies’ conception of sexuality is unique to their culture [1, 2]. However, in today's world, where social networks and global communications are critical components of any societal context, informal sexual relationships (ISR), as a risky sexual behavior, are becoming more prevalent in more reserved cultures, such as that in Iran [3, 4]. ISR is problematic since it predisposes individuals to sexually transmitted infections such as HIV [5, 6].

In parallel with globalization, Iran's internet user base has seen a remarkable increase from 250,000 in December 2000 to 67,602,731 in April 2021, placing the country among the top 20 countries with the highest number of internet users [7]. We hypothesize that because access to the internet and various virtual networks is a significant factor in decision-making nowadays, and since people spend more time online every day, risky sexual behaviors are associated with free internet content [8, 9].

Smartphones will expose users to explicit sexual content through a variety of means, including access to websites, the growth of dating apps whose primary purpose is not mating, but rather befriending a potential partner, and the ability to locate a sexual partner through these apps [10–12]. Furthermore, a more implicit aspect attributed to this issue is the effect of social media on this impressionable generation, influencing young adults' offline sexual behaviors [13, 14].

It is critical to understand the role of religiosity when discussing sexual behavior in countries with a religious context. Since extramarital sexual relations are considered as a major sin in some religions, they instill feelings of guilt in those who violate these religions' laws, and this mechanism ostensibly deters people from committing this act [15, 16]. Moreover, having ISRs and engaging in risky sexual behaviors are affected by personality traits [17], how lonely young individuals think they are [18], and how close their parents are [19, 20].

A systematic review and meta-analysis study showed that there is a relationship between personality and sexual behaviors. Based on the results Extraversion personality was positively related to sexual activity and risky sexual behavior. Also, Openness personality was positively related to homosexual orientation [17].

Considering the multifactorial nature of informal sexual relationship and mobile app use in Iran on the one hand, and shortage of similar studies to clarify this issue in the Iranian context on the other hand, the objective of this study was determining the prevalence of ISR and its related factors in Shiraz, southwest of Iran. Obviously, the results of this study will help health and social policymakers to address its consequences and design evidence-based preventive strategies.

## Methods

### Study setting and participants

This cross-sectional study was conducted on 414 adults in their 20’s and 30’s in Shiraz, Fars province, south of Iran in 2019. Data was collected via a web-based structured questionnaire (designed by a software engineer) which was available online. The participants needed to sign an informed consent form before submitting the questionnaire. The inclusion criterion was using smartphone apps and the questionnaire was distributed on different social media platforms, such as WhatsApp, Telegram, and Instagram.

### Data collection

Through the questionnaire, we collected data concerning demographic variables, namely age, sex (male, female), birthplace (province capital, small cities, and rural areas), marital status (single or married), educational background (high school, diploma, associate degree, bachelor’s, post graduate), socio-economic status (low, middle, high). The smartphone use-related variables were also investigated, such as the duration of having a smartphone (in years), duration of internet connection in day (in hours), number of social media apps, and whether they use these apps to find a friend and have sexual relations.

The participants were asked to answer two five-point Likert questions regarding their religiosity and their relationship with their parents and an open question about whether they are sexually active (having sex with her/his partner) or not. The religious question: “I am a believer and religious person” with the answers: completely agree, agree, somewhat agree, disagree, and completely disagree. The question of the close relationship with the family: “My relationship with one or both of my parents is close and friendly” the answers: completely agree, agree, somewhat agree, disagree, and completely disagree.

In this study, the dependent variable was having ISR (having pre-marital sexual relationships for the single participants or having extra-marital sexual relationships for the married ones). The dependent variable was coded as yes (code 1) for those who reported having ISR, and no (coded 0) for those who reported not having ISR.

### Measurements

In this study, a validated of the short-form of UCLA Loneliness Scale (ULS-6) was used to assess loneliness. The ULS-6 is constituted by six items of the UCLA that five items are formulated in a negative way, and one in a positive way. All the six items were scored on a four-point scale ranging from 1 (never) to 4 (often), with higher scores indicating greater loneliness [21]. Zarei et al. validated the UCLA Loneliness Scale in Iran and reported adequate validity and reliability of this questioner. The intraclass correlation coefficient (ICC) for the total loneliness scores was 0.93. Also, Cronbach’s alpha was 0.91 for the total items [22].

The ten-item personality inventory (TIPI) was employed to measure the Big Five personality domains (extraversion, agreeableness, conscientiousness, emotional stability, openness to experiences). Each of the Big Five dimensions are measured with two items. All the items were scored on a seven-point scale ranging from 1 (strongly disagree) to 7 (strongly agree) [23]. Azkhosh et al. validated the TIPI in Iran and reported adequate validity and reliability of this questioner. Cronbach’s alpha of 0.51 was found for the TIP. Also, ICC for the total TIPI was 0.92 [24].

### Statistical analysis

The quantitative variables were described as mean ± standard deviation and the qualitative ones were reported as numbers and percentages. Simple and multiple backward logistic regression models were used for analyzing the ISR-associated factors. The variables with *p* ≤ 0.2 in the simple logistic regression analysis were considered to be included in the multiple logistic regression analysis and tested by backward elimination. Odds ratio (OR and 95% CI) was reported as the indicator of the association between the independent variables and the dependent one. A *p* value ≤ 0.05 was considered to be significant. Statistical analysis was performed with Statistical Package for Social Sciences (SPSSs) version 20.0 and Graph Pad Prism software, version 9.0.

### Ethical considerations

Ethical issues (including plagiarism, informed consent, misconduct, data fabrication and/or falsification, double publication and/or submission redundancy) have been completely observed by the authors. The ethical approval for this study was provided by the Ethics Committee at Shiraz University of Medical Sciences under the ethical code of IR.SUMS.REC.1397.112.

## Results

### Demographic characteristics of the participants

**Fig. 1:**
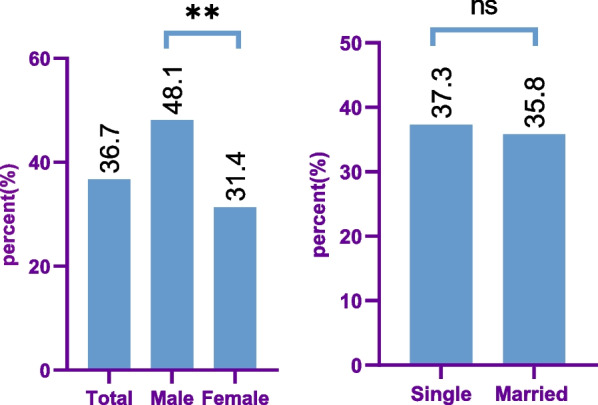
Prevalence of ISR by sex and marital status

Among the 414 participants recruited herein, 283 (68.4%) were female. The mean age of the participants was 27.5 ± 6.5 years. Regardless of their marital status, 152 (36.7%; 95% CI 32.1–45.6) had ISR, comprising 89 (31.4%) females and 63 (48.1%) males; therefore, ISR was significantly more frequent in males. It was also higher among the single subjects (37.3%) compared to the married ones (35.8%); however, this difference was not statistically significant (Fig. 1). The results of the socio-demographic characteristics according to having ISR or not are represented in Table 1.

**Table 1:** Characteristics of participants according to having or not having ISR

Variables	Having ISR (n = 152)	Not having ISR (n = 262)
Age	27.85 ± 5.93	27.33 ± 6.86
Sex		
Male	63 (48.1)	68 (51.9)
Female	89 (31.4)	194 (68.6)
Marital status		
Single	90 (37.3)	151 (62.7)
Married	62 (35.8)	111 (64.2)
Education		
High school	17 (11.2)	12 (4.6)
Diploma	31 (20.4)	45 (17.2)
Associate degree	23 (15.1)	36 (13.7)
Bachelors	47 (30.9)	112 (42.7)
Post graduate	32 (21.1)	55 (21.0)
Birth place		
Capital city	121 (79.6)	141 (53.8)
Small city	19 (12.5)	96 (36.6)
Rural area	12 (7.9)	25 (9.5)
Socioeconomic status		
Low	26 (17.2)	56 (21.5)
Middle	84 (55.6)	134 (51.3)
High	41 (27.2)	71 (27.2)
Duration of connecting to the Internet		
Less than 1 h per week	2 (1.3)	4 (1.5)
1–5 h per week	7 (4.6)	10 (3.8)
Less than 1 h per day	7 (4.6)	18 (6.9)
1–3 h per day	33 (21.7)	80 (30.5)
3–5 h per day	26 (17.1)	58 (22.1)
More than 5 h per day	77 (50.7)	92 (35.1)
Intimate relationship with parents		
Not intimate	86 (57)	178 (67.9)
Somewhat intimate	40 (26.5)	69 (26.3)
Intimate	25 (16.6)	13 (5.0)
Number of mobile apps		
One	5 (3.3)	17 (6.5)
Two	33 (21.7)	59 (22.5)
Three	51 (33.8)	108 (41.2)
More than three	62 (40.8)	78 (29.8)
Duration of having a smartphone		
Less than 1 year	1 (0.7)	7 (2.7)
1–2 years	10 (6.6)	29 (11.1)
3–4 years	43 (28.3)	86 (32.8)
More than 4 years	96 (63.2)	139 (53.1)
Find a friend through a mobile app		
Yes	68 (44.7)	48 (18.3)
No	84 (55.3)	214 (81.7)
Currently sexually active		
Yes	79 (52.0)	91 (34.7)
No	73 (48.0)	171 (65.8)
Loneliness	14.81 ± 4.29	13.67 ± 4.57
Personality domains		
Agreeableness	9.97 ± 244	10.46 ± 2.34
Extraversion	8.04 ± 2.37	6.98 ± 2.52
Consciousness	10.89 ± 2.71	11.23 ± 2.41
Openness to experiences	9.05 ± 2.39	7.55 ± 2.55
Emotional stability	8.27 ± 2.76	8.92 ± 2.85
Religious beliefs		
Strongly disagree	33 (21.7)	15 (5.7)
Disagree	24 (15.8)	25 (9.5)
Medium	64 (42.1)	89 (34.0)
Agree	23 (15.1)	59 (22.5)
Strongly agree	6 (3.9)	72 (27.5)

Qualitative variables are reported as number (percentage) and quantitative variables as mean ± standard deviations

### Factors associated with ISR: simple model

According to the results of simple logistic regression, the following factors were found to be significantly associated with ISR: male sex (OR = 2.02; 95% CI 1.32, 3.09; *p* value: 0.001), longer duration of internet use (OR = 1.21; 95% CI 1.02, 1.43; *p* value: 0.029), further closer relationship with parents (OR = 3.98; 95% CI 1.94, 8.16; *p* value: 0.001), high duration of having a smartphone (OR = 1.45; 95% CI 1.08, 1.94; *p* value: 0.013), finding an opposite-sex friend through a mobile app (OR = 3.61; 95% CI 2.31, 5.64; *p* value: 0.001), being currently sexually active (OR = 2.03; 95% CI 1.35, 3.06; *p* value: 0.001), loneliness (OR = 1.06; 95% CI 1.01, 1.11; *p* value: 0.014), having an extroverted personality (OR = 1.17; 95% CI 1.07, 1.27; *p* value: 0.001), and being open to new experiences (OR = 1.27; 95% CI 1.17, 1.39; *p* value: 0.001). Furthermore, a high level of education, living in a small city rather than a capital city, having religious beliefs, having an agreeable personality, and high emotional stability had a negative association with having ISR; these were protective factors in terms of having ISR (Table 2).

**Table 2:** Factor associated with ISR; simple analysis

Variables	OR	95% CI*	*p* value
Age	1.02	0.98–1.04	0.438
Sex			
Female	Ref.	–	–
Male	2.02	1.32–3.09	0.001*
Marital status			
Single	Ref.	–	–
Married	0.94	0.62–0.1.41	0.754
Education	0.83	0.70–0.97	0.024*
Birth place			
Capital city	Ref.	–	–
Small city	0.23	0.13–0.39	0.001*
Rural area	0.56	0.27–1.16	0.119
Socioeconomic status	1.15	0.88–1.51	0.297
Duration of connecting to the Internet	1.21	1.02–1.43	0.029*
Intimate relationship with parents			
Not intimate	Ref.	–	–
Somewhat intimate	1.20	0.75–1.91	0.444
Intimate	3.98	1.94–8.16	0.001*
Number of mobile apps	1.23	0.97–1.55	0.075
Duration of having a smartphone	1.45	1.08–1.94	0.013*
Find a friend through a mobile app	3.61	2.31–5.64	0.001*
Currently sexually active	2.03	1.35–3.06	0.001*
Loneliness	1.06	1.01–1.11	0.014*
Personality domains			
Agreeableness	0.92	0.84–0.099	0.048*
Extraversion	1.17	1.07–1.27	0.001*
Consciousness	0.14	0.87–1.03	0.196
Openness to experiences	1.27	1.17–1.39	0.001*
Emotional stability	0.92	0.86–0.99	0.026*
Religious beliefs			
Strongly disagree	Ref.	–	–
Disagree	0.43	019–0.99	0.050
Medium	0.33	016–0.65	0.001*
Agree	0.17	0.08–0.38	0.001*
Strongly agree	0.04	0.01–0.10	0.001*

*CI* confidence interval

*Significant at 0.05 level

### Factors associated with ISR: multiple model

In multivariate analysis, as shown in Table 3, finding an opposite-sex friend through a mobile app (OR = 2.59; 95% CI 1.34, 5.01; *p* value: 0.005), being currently sexually active (OR = 2.39; 95% CI 1.26, 4.56; *p* value: 0.008), having an extroverted personality (OR = 1.13; 95% CI 1.01, 1.27; *p* value: 0.029), and further closer relationship with parents (OR = 3.17; 95% CI 2.25, 8.02; *p* value: 0.015) were associated with having ISR. Moreover, living in a small city was indicated as a protective factor for having ISR.

**Table 3:** Factor associated with ISR; Multiple analysis

Variables	OR	95% CI	*p* value
Sex			
Female	Ref.	–	–
Male	0.88	0.46–1.71	0.724
Birth place			
Capital city	Ref.	–	–
Small city	0.23	0.10–0.49	0.001*
Rural area	0.64	0.22–1.81	0.398
Find a friend through a mobile app	2.59	1.34–5.01	0.005*
Currently sexually active	2.39	1.26–4.56	0.008*
Extraversion personality	1.13	1.01–1.27	0.029*
Intimate relationship with parents			
Not intimate	Ref.	–	–
Somewhat intimate	1.04	0.53–2.01	0.916
Intimate	3.17	2.25–8.02	0.015*

*CI* confidence interval

*Significant at 0.05 level

## Discussion

The results of our study revealed that approximately 36% of the participants had experienced ISR, which was associated with increased duration of internet use, duration of having a smartphone, and finding an opposite-sex friend through a mobile app. Also, residency area is a significant determinant for ISR, which means that living in small town associated with less ISR compared to the large cities. Moreover, findings of this study showed that the participants' personality including agreeableness, extraversion, and openness to new experiences were associated with having ISR. Based on simple analysis model, another associated factor with ISR was religiosity, although in multiple analysis it was not significant. Furthermore, marital and socio-economic status did not show significant association with ISR, while the level of relationship with parents did. In fact, the closer relationship with any parents, the less involvement with ISR.

In line with the results of the present research, Choi et al. reported a relationship between ISR and prolonged use of dating apps and the internet (more than 12 months) [9]. Other studies have indicated that individuals who use these methods to identify potential sexual partners engage in more sexual activity and have a more significant number of sexual partners and more likely to have sexual interactions with someone they meet online [4–6]. One possible reason behind this issue is that internet platforms provide information that attracts a more significant number of sexually active individuals or those looking for potential partners compared to other types of people. The ability of online dating sites to provide possibilities for sexual partner-seeking users who have specific sexual preferences or are geographically separated have an important role [25]. Additionally, it has been demonstrated that people are more comfortable discussing sex online [26].

We found that living in small towns rather than large cities had a negative association with having ISR. In fact, living in small towns was a protective factor for having ISR among the participants. Alimoradi et al. reported that the geographic location of one's residence can significantly impact his or her attitude toward sexual relationships [27]. Other relevant studies have indicated that living in urban areas and having a history of sexual relations with people they meet on the internet were associated with ISR [12]. This finding could be explained by people's adherence to social traditions and their knowledge of social networks in small cities. In addition, in comparison with capital cities, small cities may have more limited access to virtual sexual partners, which could explain why the results are statistically significant when comparing capital cities to small cities [28].

We assessed the participants' personality and found that agreeableness, extraversion, and openness to new experiences were all associated with having ISR. According to our findings, the individuals with higher scores concerning consciousness and emotional stability were less likely to participate in ISRs. A meta-analysis of the association between the Big Five personality traits and sexual behavior showed that openness to new experiences was positively associated with liberal attitudes toward sexual relations. On the contrary, agreeableness and conscientiousness were adversely associated with the traits described previously. Extraversion and a desire to try new things were found to be associated with more liberal views on sexual relationships in this study [17].

Based on our results, stronger religious beliefs had a stronger protective effect on risky sexual behaviors among the participants. More robust religious beliefs had a more significant protective effect against unsafe sexual practices. This finding is in agreement with those of a previous paper conducted by Honarvar et al. who reported the lack of religious beliefs as a significant predictor of premarital sex [29]. Chen et al. demonstrated that weekly religious attendance compared with no attendance was related to fewer lifetime sexual partners and a lower risk of early sexual initiation [30].

Additionally, our study revealed that the individuals with a higher level of education had a lower risk of developing ISR. Contrariwise, Carlos et al. discovered a link between superior education and various relationships [31]. However, when we conducted multiple regression analyses, there was no association between education and ISR. This inconsistency could be due to different study populations or other confounding factors in each study.

According to the obtained results herein, there was no statistically significant association between marital status and having an ISR. Nonetheless, Cabecinha et al. reported that women without a stable partner were more likely to report using the internet to find sexual partners whereas women with stable spouses were less likely to report using the internet to find sexual companions [32]. According to another study, having ISR could be further linked to a dating relationship (rather than a long-term relationship) [9].

In the present study, ISR was not associated with socioeconomic status. A study conducted by Watchers Smith et al. implied that men's history of sex with someone they have met on the internet was not significantly associated with their annual household income [12]. Another study reported that people with low socioeconomic status have poorer health outcomes and are more likely to contract a sexually transmitted infection [33].

Finally, we found that an increased risk of developing ISR was associated with a closer relationship with one's parents. Similarly, another study reported that lax parental monitoring enhances adolescents' risky sexual behaviors [34]. This association is most likely due to parental intimacy and parenting styles. A possible interpretation would be that, parental intimacy is not always indicative of family ideals. We need to do further study and delve into the parenting strategies used by people who had close relationships with their parents and engaged in riskier sexual behaviors to find the logic behind it.

As previously stated, this study is one of the few studies exploring the factors associated with ISR among Iranian young adult’s smartphone users. However, our study has certain limitations. Regarding the sensitive and intimate nature of the study, the respondents' answers would be biased even though the scientists attempted to mitigate this bias using an online and anonymous questionnaire. Moreover, due to socio-political barriers and limited literacy in using web- based applications we could not increase the sample size more than 414. Therefore, the findings of this study may not be generalizable to all the people in this age group despite the researchers' best attempts to ensure the maximum diversity of responders by sending the questionnaire's link to various clusters of the population nationwide.

## Conclusion

In conclusion, the present study illustrated the prevalence of Informal Sexual Relationship amongst individuals in the age range of 20–40 in Shiraz and its association with the increased duration of internet and mobile app use. In view of this social phenomenon, innovative and multidisciplinary approaches that focus on predictive and protective factors in community settings and intervention strategies for behavioral and legislative changes are required. Further research could be suggested to evaluate the confounding factors in this regard.

## Data Availability

The datasets used and/or analyzed during the current study available from the corresponding author on reasonable request.
